# Somatic mutation landscape in a cohort of meningiomas that have undergone grade progression

**DOI:** 10.1186/s12885-023-10624-9

**Published:** 2023-03-07

**Authors:** Sarah A Cain, Bernard Pope, Stefano Mangiola, Theo Mantamadiotis, Katharine J Drummond

**Affiliations:** 1 Department of Neurosurgery, The Royal Melbourne Hospital, 300 Grattan street, Parkville, VIC Australia; 2 Melbourne Bioinformatics, The University of Melbourne, Parkville, Australia; 3 The Walter and Eliza Hall Institute of Medical Research, Parkville, Australia; 4 Department of Microbiology & Immunology, The University of Melbourne, Parkville, Australia; 5 Department of Surgery (Royal Melbourne Hospital), Melbourne Medical School, Faculty of Medicine, Dentistry and Health Sciences, The University of Melbourne, Melbourne, Australia; 6 Department of Medicine, Central Clinical School, Faculty of Medicine Nursing and Health Sciences, Monash University, Monash, Australia

**Keywords:** Meningioma, Next generation sequencing, Anaplastic, Atypical, Malignant

## Abstract

**Background:**

A subset of meningiomas progress in histopathological grade but drivers of progression are poorly understood. We aimed to identify somatic mutations and copy number alterations (CNAs) associated with grade progression in a unique matched tumour dataset.

**Methods:**

Utilising a prospective database, we identified 10 patients with meningiomas that had undergone grade progression and for whom matched pre- and post-progression tissue (n = 50 samples) was available for targeted next-generation sequencing.

**Results:**

Mutations in *NF2* were identified in 4/10 patients, of these 94% were non-skull base tumours. In one patient, three different *NF2* mutations were identified in four tumours. *NF2* mutated tumours showed large-scale CNAs, with highly recurrent losses in 1p, 10, 22q, and frequent CNAs on chromosomes 2, 3 and 4. There was a correlation between grade and CNAs in two patients. Two patients with tumours without detected *NF2* mutations showed a combination of loss and high gain on chromosome 17q. Mutations in *SETD2*, *TP53*, *TERT* promoter and *NF2* were not uniform across recurrent tumours, however did not correspond with the onset of grade progression.

**Conclusion:**

Meningiomas that progress in grade generally have a mutational profile already detectable in the pre-progressed tumour, suggesting an aggressive phenotype. CNA profiling shows frequent alterations in *NF2* mutated tumours compared to non *NF2* mutated tumours. The pattern of CNAs may be associated with grade progression in a subset of cases.

**Supplementary Information:**

The online version contains supplementary material available at 10.1186/s12885-023-10624-9.

## Background

Meningiomas are the most common primary intracranial tumours, accounting for over 35% [1]. The World Health Organisation (WHO) Classification of Central Nervous System (CNS) Tumours, 2016 revision, classifies meningiomas into Grade 1 (benign), Grade 2 (atypical), or Grade 3 (malignant or anaplastic). Although the majority are Grade 1 and surgically curable, Grade 2 and 3 tumours account for 5-15% and 1-3%, respectively. Meningiomas are characterised by histological and biological diversity, with 15 different WHO histopathological subtypes.

Mainstay treatment for Grade 2 and 3 meningiomas is surgical resection. Post-operative radiotherapy is standard for Grade 3 tumours, but controversial for Grade 2 tumours and is the subject of an ongoing international multicentre randomised trials [2, 3]. Grade 2 and 3 meningiomas frequently recur and are associated with significant morbidity and mortality. 30-40% of Grade 2 meningiomas recur, increasing to 50-80% for Grade 3 [4]. Despite excellent overall 5-year survival for Grade 1 meningiomas, rates decrease to 78% for Grade 2 and 44% for Grade 3 [5].

Recent studies have expanded our understanding of the genetic and epigenetic landscape of meningiomas. *NF2* is the most commonly involved gene, with up to 60% of sporadic meningiomas exhibiting biallelic mutation or loss of the *NF2* tumour suppressor gene on chromosome 22 [6]. The remaining 40% show a variety of gene mutations, often in combination, including *TRAF7, KLF4, AKT1, POLR2A, PIK3CA, SMO, TERT, SMARCE1*, *TP53, BAP1, CDKN2A, CDKN2B* and *mTOR*.

Interestingly, the intracranial or spinal location of meningiomas is associated with distinct genetic profiles. Grade 3 *NF2* mutant meningiomas are most commonly parasagittal, falcine, torcular or intraventricular. Secretory meningiomas are preferentially located at the anterior skull base and always harbour a *KLF4* mutation, which is usually coexistent with mutations in *TRAF7*, whereas *AKT1* and *SMO* mutations are enriched in the meningothelial subtype and tumours of the anterior midline skull base [7–10]. Both *PIK3CA* and *POLR2* mutations predispose to anterior and middle skull base meningiomas [7–10]. *NF2, TERT, SMARCB1, SMARCE1* and *BAP1* have been associated with higher grade meningioma and poor prognosis [11].

Meningiomas that progress in WHO grade require multiple surgeries, often with increasing morbidity. Grade progression is associated with an increased incidence of recurrence [12]. Key to reducing morbidity is understanding the molecular drivers of progression to expand treatment options beyond surgical resection and radiotherapy. Previous studies have partially elucidated changes associated with grade progression, including genomic instability, loss of the *CDKN2A*/*CDNK2B* locus on chromosome 9q and deletion of chromosome 1p [13, 14]. Patel et al. sequenced 128 meningiomas which included three patients reported to have meningiomas that underwent grade progression exhibiting the most genomic instability, predominantly loss of chromosome 22q and 1p [15].

We previously reported a 17% histopathological progression rate in a dual centre Grade 2 and 3 meningioma cohort [16]. From this cohort we have identified a unique subset of patients with matched tissue samples (pre- and post-progression). Utilising this unique cohort, we aimed to identify genetic drivers of pathological progression using targeted next-generation sequencing.

## Methods

Approval was granted by the Human Research Ethics Committee (HREC) at The Royal Melbourne Hospital (HREC 2017.288). The Australian Comprehensive Cancer Outcomes and Research Database (ACCORD) is a multi-institutional database of cancer patient demographics, treatment and clinical outcomes managed by BioGrid Australia [17]. It includes longitudinal data on multiple cancers. The CNS Tumour Database, from this group of datasets, prospectively enrols and follows all patients with CNS tumours treated at the Royal Melbourne Hospital (RMH) and the co-located Melbourne Private Hospital (MPH). This database was interrogated to identify patients with WHO Grade 2 and 3 meningioma enrolled since database commencement in 2009.

### Patient selection and data Collection

The CNS Tumour Database search undertaken for our previously published cohort identified 67 patients with Grade 2 and 3 meningiomas resected at RMH/MPH and entered prospectively over 4 years. Of these, nine (16%) were excluded; six (9%) due to previous resections at other institutions or with destroyed records and three (4%) were lost to follow-up. Of the remaining 58 patients, 10 were identified who had tumours that had progressed in WHO Grade and had matched samples available for analysis.

Patient demographics (sex, date of birth, baseline Eastern Cooperative Oncology Group [ECOG] performance status), tumour characteristics (WHO grade, tumour location, radiology date), surgical details (type of surgery, extent of resection), and adjuvant therapy (chemotherapy, radiotherapy – date, dose and fractions) were collated. Extent of resection was designated as gross total resection (GTR, > 95%), subtotal resection (STR, 50–95%) or partial resection (PR, < 50%), as confirmed by both the surgeon’s impression at the time of surgery (100%) and first post-operative imaging (80% available).

Backfill of data allowed tumours to be included in the analysis that were previously resected as early as 1989. Central pathology review by an experienced neuropathologist was performed based on the WHO Classification, 2016 revision, to ensure consistency of grading. As a result of this review, one patient’s tumour was reclassified to Grade 2 from Grade 3.

### Tissue selection and processing

Formalin fixed paraffin embedded (FFPE) tissue was retrieved from the Royal Melbourne Hospital Department of Anatomical Pathology. Patients were deidentified and assigned letters A to J. Tumours from each patient were numbered non-sequentially to avoid bias. A 4 μm section from each block was stained with hematoxylin and eosin (HE) and examined by a neuropathologist to ensure tumour purity, determine cellularity and exclude necrosis. Three further consecutive 10 μm sections were cut from each block and used for DNA extraction. Four patients had duplicate tumour samples sent for quality control, the bioinformatics analysis was blinded to this to exclude bias.

### DNA extraction and quality assessment

Tumour cell rich regions, identified by examination of the HE-stained sections and matching the regions on the 10 μm consecutive tissue sections, were micro-dissected from three slides, and same-specimen samples were pooled, and DNA extracted using Qiagen DNA deparaffinisation solution, followed by processing using a Qiagen QIAamp DNA FFPE Tissue Kit (Qiagen, Germantown, MD, USA). The sample yield, purity and quality of the eluted DNA were assessed using a Qubit dsDNA BR Assay Kit and Qubit Fluorometer (ThermoFisher, Waltham, MA, USA) and the Agilent QC-Plex multiplex PCR assay (Agilent, Santa Clara, CA, USA), respectively. For samples to be sequenced, DNA yield was at least 40ng per sample and DNA Quality Coefficient (DQC), as determined by Agilent Qcplex, was at least 0.34. Sample DCQ ranged between 0.34 and 1.88.

### Next generation sequencing

Ten microlitres of each normalised DNA library were pooled and incubated at 96 °C for 2 min. The library pool solution was mixed, centrifuged briefly and incubated on ice for 5 min. Libraries were sequenced on an Illumina Nova-Seq instrument. DNA libraries were prepared using the hybrid capture-based TruSight Oncology 500 (TSO500) Library Preparation Kit (Illumina, San Diego, CA, USA) following Illumina’s TSO500 reference guide targeting 523 cancer-relevant genes with a target capture size of 1.94 megabases (Mb) (see supplementary data 2 for a list of genes). The panel includes the following Meningioma-related genes and targets from the literature: *NF2*, *KDM5C*, *KDM6A*, *SMARCB1*, *AKT1*, *mTOR*, *SMO*, *TRAF7*, *KLF4*, *PIK3CA*, *BAP1*, *TP53*, *CDKN2A*, *CDKN2B*, *TERT* (+ promoter), *NAB2*-*STAT6* fusion. Mean sequencing yield in the TSO500 data was 18 gigabases (Gb) per sample, with a mean unfiltered depth of coverage of 9278 reads per sample.

A subset of 13 tumours was also sequenced using the Agilent SureSelect Clinical Research Exome V2 (Agilent, Santa Clara, CA, USA) with a capture size of 67.3 Mb. Mean sequencing yield in the exome data was 46 Gb per sample, with a mean unfiltered depth of coverage of 688 reads per sample.

### Bioinformatic analysis

Sequencing reads for the TSO500 and exome data were assessed using FastQC (v0.11.9) [18]. All samples were deemed to be sufficiently high quality for further analysis. Sequencing reads were aligned using BWA (v0.7.17) [19] to the GRCh38 human genome reference from the GATK resource bundle downloaded from the Broad Institute on 1 February 2021. Depth of sequencing was assessed across target regions using Mosdepth (v0.3.1) [20]. The sex of each sample was confirmed by the coverage observed on the X and Y chromosomes. Sequencing read deduplication using unique molecular identifier barcodes (UMIs) was performed using Connor (v0.6.1) [21] for the TSO500 panel and Gencore (v0.13.0) [22] for the exome data.

In both datasets, putative single nucleotide variants (SNVs), short insertions and deletions (indels) and structural variants (SVs) were called using VarDict (v1.8.2) [23]. Variants were then annotated for their predicted effect, gnomAD (v3.1.1) [24] population frequency and ClinVar [25] pathogenicity using VEP (v101) [26], and genes affected by mutations were annotated for their presence in Tiers 1 and 2 of the COSMIC Cancer Gene Census [27] (downloaded from the COSMIC website on 14 February 2021). The putative set of variants was filtered to a high-confidence set of likely somatic mutations as follows. Synonymous SNVs were removed because they are unlikely to have a functional effect. A minimum depth of coverage of 50 reads was required to remove regions with low-quality mapping to the reference genome. Variants with a variant allele fraction (VAF) < 0.1 were removed to avoid likely FFPE-induced noise and sequencing artefacts, and variants with a VAF > 0.9 were removed to avoid likely germline homozygous variants. Variants with a gnomAD population frequency > 0.0001 (one in ten thousand) were removed to avoid likely germline polymorphisms. Variants that remained after filtering were annotated with their frequency within the data set (separately for TSO500 and exome), with the expectation that a higher study cohort frequency is inversely proportional to the likelihood of being associated with a meningioma pathogenicity (highly recurrent variants across patients are likely to be either unfiltered germline polymorphisms or artefacts). High-confidence, likely pathogenic variants identified in the TSO500 data were visually inspected in the TSO500 sequencing alignment (BAM) files and confirmed in the exome data for tumours where both types of sequencing were performed and the capture regions containing the variants were overlapping (notably, *TERT* promoter regions were present in the TSO500 capture, but not in the exome).

Copy number alterations on autosomal chromosomes were identified using a combination of per-gene sequencing depth log-fold-change (LFC, also called log R ratio) and the b-allele-frequency (BAF) of single nucleotide polymorphisms (SNPs). Specifically, LFC was computed for each gene in each sample by taking log_2_ of the ratio of the median depth of coverage in the gene and the median depth of coverage in the tumour sample. In this method, the median depth of the tumour sample is used to estimate the dominant baseline copy number, and any significant divergence away from the median indicates copy gain or loss for a gene relative to the baseline. SNPs from the 1000 Genomes Phase 1 High Confidence dataset [28] (downloaded from the Broad Institute on 1 February 2021) were intersected with the TSO500 target coordinates yielding 13,294 loci. The BAF was calculated at each of these loci as the proportion of all aligned reads supporting the non-reference allele. A BAF close to 0.5 indicates a heterozygous site (HET), while values close to 0 or 1 indicate homozygous sites (HOM). BAF deviations away from HET and HOM states indicate sites of allelic imbalance, such as copy loss and gain or copy-neutral loss of heterozygosity (LOH). In order to identify regions of copy number change, both the LFC and BAF values for each tumour sample were independently clustered using a Gaussian mixture model (GMM). In each case, the best fitting cluster model was determined by searching for the number of clusters that minimised the Bayesian information criteria (BIC). For each tumour sample, the LFC and BAF clustered data was plotted in genomic coordinate order and CNAs were determined by visual inspection of both plots. Copy number changes were classified into one of six types: “LOH” for copy neutral loss of heterozygosity; “gain/loss” for copy number increase or decrease affecting >= 1/3 of a chromosome arm; “focal gain/focal loss” for copy number increase or decrease affecting <= 1/3 of a chromosome arm (usually a few genes only); and “aberrant” for circumstances where the LFC and/or BAF indicated clear deviation from the heterozygous state, but the data could not be unambiguously assigned to a single type of copy number change (for example, perhaps due to subclonality). For the 13 tumours having both TSO500 and WES sequencing data, CNAs identified in the TSO500 data were verified in the corresponding WES data. The highly recurrent nature of CNAs across tumours from the same patient was also used to corroborate the calls.

To evaluate the association between genomic aberrations (e.g. mutations and fusions) and gene copy number changes, we performed a quasibinomial regression with the glm (generalized linear model) R package. We dichotomised the patients based on having a specific genomic aberration, and tested the difference in copy number change (irrespective of the type). To avoid strong correlations among samples from the same patient we averaged the binary copy number event (1 = with copy number change, 0 = without copy number change). We corrected the p-values for multiple testing with the Benjamini and Hochberg method (supplementary data 3) [29].

## Results

**Fig. 1:**
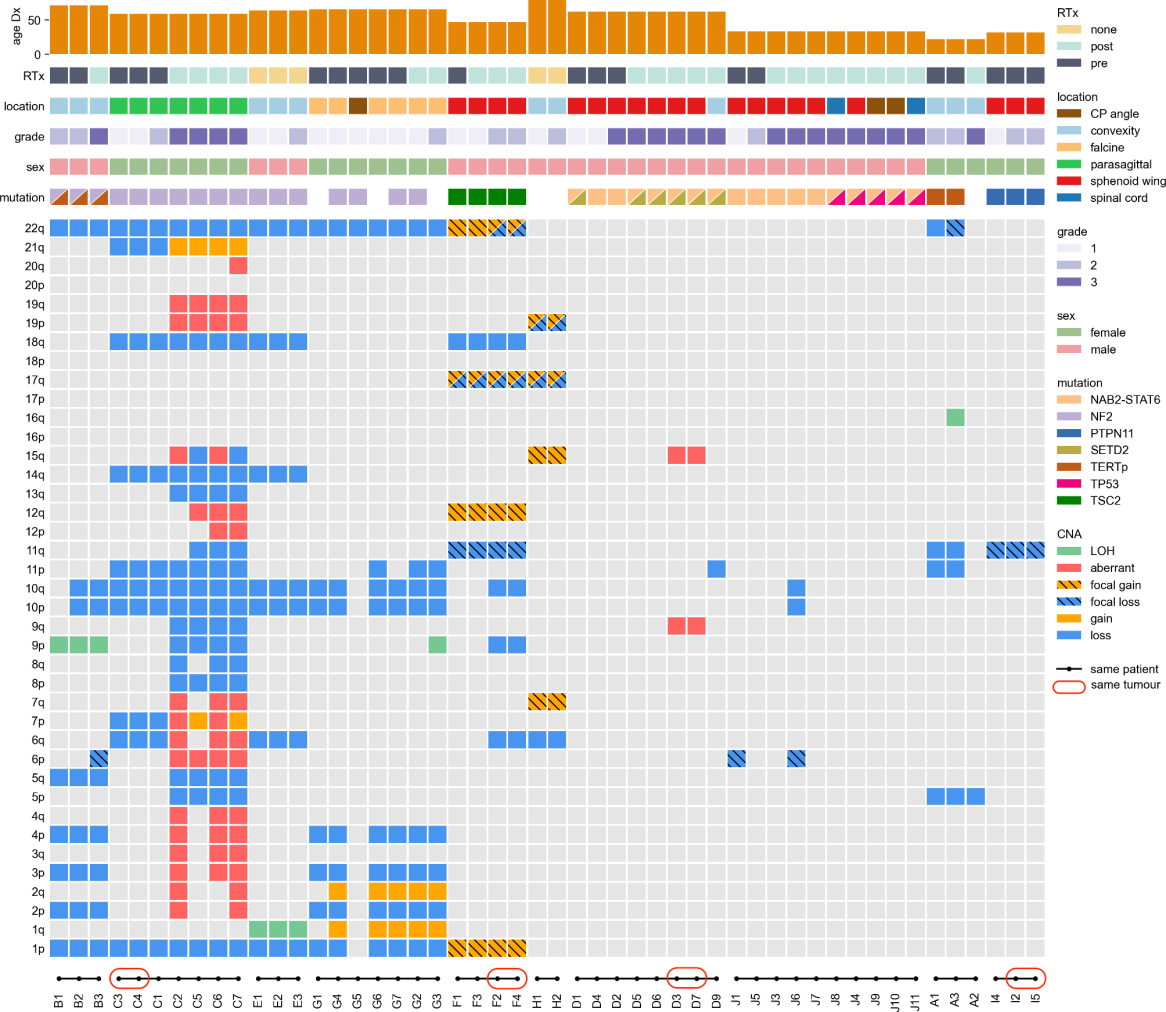
**Landscape Plot** Summary of CNA and mutations across all sequenced tumours. Radiotherapy (RTx), post = post resection, pre = pre resection. Cerebellopontine (CP). Grade refers to WHO grading. Loss of Heterozygosity (LOH). Red circle highlights the same tumour which was blinded for quality control

An overall summary of patient demographics, tumour samples, significant somatic mutations and copy number alterations is provided in Fig. 1.

### Patient demographics

Ten patients were included in the final analysis (Table 1). At initial diagnosis, seven patients had Grade 1 tumours and three Grade 2. During follow-up five patients had tumours that progressed to Grade 3 with the remaining five progressing to Grade 2.

**Table 1:** Baseline Characteristics at Diagnosis

Patient	Age at Diagnosis	Sex	ECOG	Year Diagnosed	WHO Grade	Location*	Number of resections	Adjuvant Radiation*	Status
*A*	22	F	0	2005	2 → 3	Convexity	3	Yes	Alive
*B*	71	M	1	2000	2 → 3	Convexity	3	Yes	Died 2003
*C*	59	F	0	1997	1 → 3	Parasagittal	6	Yes	Died 2008
*D*	62	M	2	2000	1 → 3	Sphenoid Wing	7	Yes	Died 2017
*E*	64	M	1	2003	1 → 2	Convexity	3	No	Alive
*F*	47	M	0	1997	1 → 2	Sphenoid Wing	3	Yes	Died 2007
*G*	65	F	1	1989	1 → 2	Falcine CP angle	7	Yes	Died 2004
*H*	79	M	1	2000	1 → 2	Convexity	2	No	Died 2006
*I*	32	F	0	2006	1 → 2	Sphenoid Wing	3	Yes	Alive
*J*	33	M	0	1997	2 → 3	Sphenoid Wing CP angle Spinal Cord	10	Yes	Died 2009
	**53.4**	**2:3**					**5**	**80%**	

*Details of radiation timeline, relationship tumour location across resections and timing of progression is detailed in the supplementary information. CP = cerebellopontine

Mean age at diagnosis was 53 years (range 22–79) and there was a male-to-female ratio of 2:3. The majority of patients presented with an excellent performance status, with 90% having an ECOG Grade 0 or 1. All patients underwent multiple resections; two (one patient), three (five patients) and more than three (four patients). One patient had ten operations in total. Overall, the GTR rate at initial resection was 80%. 80% of patients received radiation, the timing of which in relation to surgery is demonstrated in Fig. 1.

### Variant calling

A pathogenic *NF2* mutation was detected in at least one tumour in four (40%) patients (B, C, E, G) (Fig. 1). Two of these patients had tumours located at the convexity (B, E), one was parasagittal (C) and one was falcine (G). Patient G had four tumours with three different *NF2* somatic mutations, all of which were present in the pre-progressed tumours. However, the progressed tumour from this patient did not show a *NF2* mutation. One tumour from patient G had two *NF2* variants co-occurring on the same sequencing reads suggestive of a complex variant. In patients B, C and E an *NF2* mutation was present in all tumours (pre- and post-progression). An *NF1* mutation was also detected in all tumours from patient B.

A *TERT* promoter variant was detected in two patients (A, B), both with convexity meningiomas. *TERT* promoter variants were identified in two Grade 2 tumours from patient A, but were absent from the tumour that had progressed to Grade 3. In patient B the *TERT* promoter variant was detected in all tumours. A *TP53* variant was detected in one patient (J) with Grade 3 tumours in two different cranial locations, but not in the initially resected Grade 1 and 2 tumours (Fig. 1). A *SETD2* variant was detected in 75% of tumours from one patient (D). This patient had a skull base tumour and also had a *NAB2-STAT6* inversion mediated fusion (in all tumours), as did patient J with skull base and spinal cord tumours. Table 2 shows a list of additional mutations detected. Patients (C, D, F and I) had one tumour each that was sequenced twice; in all cases our variant calls and CNAs were identical between pairs.

**Table 2:** TSO-500 Variant Calling Summary

Patient	Initial WHO grade	Final WHO grade	Location	Variant	Chromosome
*A*	2	3	Convexity	*TERT* promoter *ERCC1*	5 19
*B*	2	3	Convexity	*NF2*:p.Phe94Ter *TERT* promoter *SPTA1* *NF1*	22 5 1 17
*C*	1	3	Parasagittal	*NF2*:p.Arg57Ter *POLE*	22 12
*D*	1	3	Sphenoid Wing	*SETD2* *NAB2-STAT6* *PIK3C2B* *DNMT3B* *ABRAXAS1* *IRF4*	3 12 1 20 4 6
*E*	1	2	Convexity	*NF2*:p.Lys332SerfsTer14 *CREBBP* *MST1* *PNRC1*	22 16 3 6
*F*	1	2	Sphenoid Wing	*TSC2*	16
*G*	1	2	Falcine CP angle	*NF2*:p.Ala323ProfsTer23 *NF2*:p.Glu342Ter NF2:p.Glu362ArgfsTer13 *SF3B1*	22 22 22 2
*H*	1	2	Convexity	*IFNGR1*	6
*I*	1	2	Sphenoid Wing	*PTPN11* *PIK3CA* *TRAF7* *LATS2* *GEN1* *LRP1B*	12 3 16 13 2 2
*J*	1	3	Sphenoid Wing CP angle Spinal Cord	*TP53* *NAB2-STAT6* *SPTA1* *SLX4* *CDK12* *NKX3-1*	17 12 1 16 17 8

### Copy number alterations

Tumours with *NF2* mutations (patients B, C, E and G) had the most similar pattern of CNAs. In contrast, tumours without a detected *NF2* mutation (patients A, D, I and J) show limited CNAs including both patients with *NAB2-STAT6* inversion (patients D and J). This trend is significant when compared with patients without *NAB2-STAT6* inversion (p = 1.97E-08). Interestingly tumours from patients F and H (*NF2* mutation not detected) share a similar pattern of CNAs, including a combination of loss and high gain on chromosome 17q, with patient F showing an four-fold coverage increase in focal CNAs including *RNF43* and *RAD51C*. *NF2*-positive patients have a genome-wide increase of gene copy-number alteration (p = 1.8E-14). Copy number losses containing the region including CDKN2A/2B were seen exclusively in the higher grade tumours from patients C (C2, C5, C6, C7) and F (F2, F4: replicates). These were not the only copy number changes from these patients that were associated with grade progression, particularly in patient C. We also observed loss of heterozygosity in this region in all tumours from patient B and the highest grade tumour from patients G (G3).

CNAs were detected across all chromosomes, but in particular chromosome 1p, 2p, 6q, 9p, 10p, 11p and 22q (Table 3). Common copy-number losses were most likely to occur in 22q (52%), 1p (37%) and 6q (26%). In addition, chromosome 10 (34%) was likely to lose fragments of its short arm 10p (16%) and its long arm 10q (20%). In comparison, common copy-number gains (CNGs) were less frequently detected, but included 17q (11%), 1q (11%) and 2q (11%). Loss of chromosome 1p was highly associated with *NF2* mutated tumours, whereas loss and gain on chromosome 17q were isolated to tumours in which no *NF2* mutation was detected.

**Table 3:** Large Scale Copy Number Changes

FEATURE	Number Patients	Number tumours	% NF2 mutant	% NF2 mutation not detected
**LOSS 1p**	4	19	100%	0%
**LOSS 10 (10, 10q)**	6	21	100%	33%
**LOSS 22.q**	5	22	100%	17%
**LOSS 9p**	3	9	75%	17%
**LOSS 2p**	3	9	75%	0%
**LOSS 3p or aberrant 3**	3	12	75%	0%
**LOSS 4p or aberrant 4**	3	12	50%	0%
**LOSS 14q**	2	10	50%	0%
**LOSS 18q**	3	14	50%	17%
**LOSS 5 (5, 5p, 5q)**	3	10	50%	17%
**LOSS 6q or aberrant 6**	4	14	50%	33%
**LOSS 11 (11p, 11q, 11, focal)**	6	20	50%	67%
**LOSS + GAIN 17q**	2	6	0%	33%

Major copy number differences in tumour grade were most prevalent in patients C and F. All Grade 3 tumours from patient C exhibited recurrent allelic imbalance across several chromosomes with BAF values suggestive of subclonal copy number changes. In patient C, all the Grade 3 progressed tumours showed duplication and LOH, not seen in the pre-progressed tumour. *TERT* copy number was associated with grade progression in patient A.

## Discussion

We performed next-generation sequencing on matched samples from 10 patients with tumours that progressed in histopathological grade. We utilised an Illumina TSO500 next-generation sequencing (NGS) panel to identify somatic mutations and differences between higher and lower-grade tumours from the same patient. We also analysed CNAs to look for patterns associated with grade progression. Over the last ten years the understanding of the genetic landscape of meningiomas has expanded greatly, but genetic drivers that explain progression in grade are poorly understood. Previous studies have examined unmatched samples from different patients, which, given the genetic heterogeneity of meningiomas, limits their usefulness. Our novel study has utilised NGS on matched tumour samples undergoing grade progression.

An understanding of the genomic changes that differentiate meningioma grades would allow identification of pathways that drive pathogenesis and grade progression, as well as the development of adjuvant therapies for these difficult-to-treat patients. The phase II trial conducted by Kaley et al. completed in 2014 has already shown that Sunitinib (a multitarget kinase inhibitor with activity against VEGF and PDGF) is active in recurrent Grade 2 and 3 meningioma patients, but there are no phase III trials registered [30]. There are ongoing phase II prospective trials for patients with recurrent or progressive meningioma include; bevacizumab monotherapy (NCT01125046), a combination of bevacizumab and electric field therapy (NCT02847559), and a third trial evaluating the activity of a single agent PD-1 inhibitor pembrolizumab (NCT03279692).

Our study reports NGS on fifty tumours confirmed to be meningiomas according to WHO 2016 classification by three separate neuropathologists. Molecular analysis revealed heterogeneity across patients, however broadly there were three groups; NF2 mutated tumours, non-NF2 mutated tumours and tumours with a NAB2-STAT6 inversion. Molecular analysis revealing a NAB2-STAT6 inversion is suggestive that despite these tumours being diagnosed as meningiomas on 17 separate neuropathology reports (7 resections in patient D and 10 resections in patient J) these tumours could be solitary fibrous tumours.

For the two patients that exhibited *NAB2-STAT6* inversions, the findings were consistent in all tumours. Gao et al. previously reported an inversion mediated gene fusion of *NAB2-STAT6* in a single patient with a Grade 3 meningioma [31]. Another study that examined immunohistochemical STAT6 nuclear staining revealed that of fifty-nine Grade 2 and 3 meningiomas tested, two Grade 3 tumours and one Grade 2 tumour were positive for STAT6, indicating that they also carried the *NAB2-STAT6* fusion [32]. On review, they were found to have morphological features of both meningioma and hemangiopericytoma, and were recategorised as ‘mesenchymal tumour not classifiable’. We performed STAT6 staining on our two patients’ samples and both were positive. Interestingly both were also negative for SSTR2a, the most reliable meningioma marker. However, it has been previously reported that although all Grade 1 and 2 meningiomas are positive for SSTR2A, this is true for only 20% of Grade 3 meningiomas [33].

All tumours with *NAB2-STAT6* gene fusion had limited CNAs unlike tumours with NF2 mutations. *NAB-STAT6* therefore appears to be a strong oncogenic driver in an otherwise stable genome, but the presence of *NAB-STAT6* fusion was seen prior to progression suggesting it is not a driver in grade progression. We acknowledge that tumours from these two patients could be molecularly reclassified as solitary fibrous tumours, this only highlights the importance of sequencing and gives credence to a shift towards molecular classification of meningiomas in line with other CNS tumours. One of the patients (D) with a *NAB-STAT6* fusion also exhibited a *SETD2* mutation. This mutation was present in the initially resected Grade 1 sphenoid wing tumour but was absent in the subsequent two resection samples (Grade 1 and 3) and present again in the subsequent four resections (Grade 3). The *SETD2* gene encodes SET domain-containing 2, a histone modifying enzyme responsible for all trimethylation of the lysine 36 residue on Histone 3 (H3K36me3). Decreases in H3K36me3 lead to alterations in gene regulation, increased spontaneous mutation frequency and chromosomal instability [34].

The involvement of the p53 tumour suppressor pathway in meningioma oncogenesis was first observed by loss of expression of the p53 protein [35]. Previous studies have shown that nuclear p53 staining was absent in all Grade 1 meningiomas, but was detected in Grade 2 and 3 tumours, with intense staining in one Grade 3 tumour with subsequent confirmed *TP53* mutation on sequencing [36]. Although smaller cohort studies have suggested that *TP53* is not likely to be involved in the aetiology of meningioma, a recent study on a large cohort of 850 high-grade/progressive meningiomas identified a group of meningiomas with poor prognosis characterised by *TERT* promoter or *TP53* mutations [37]. *TP53* mutations/overexpression have been reported in high-grade/dedifferentiated solitary fibrous tumours [38]. In solitary fibrous tumours *TP53* immunopositivity has been confirmed to be associated with malignant solitary fibrous tumours. We observed a *TP53* mutation in a Grade 3 sphenoid wing tumour in patient J that was absent in two pre-progression Grade 2 tumour samples. Notably, it was also absent in the first three tumour resections after progression to Grade 3 (supplementary data 1). The *TP53* mutation was not detected at the point of change in WHO grade, therefore we cannot confirm it as a driver in grade progression.

The protein encoded by the *TERT* gene, telomerase reverse transcriptase, contributes to cancer cell survival by maintaining telomere length and avoiding activation of cell senescence. *TERT* promoter mutations in the hotspot regions C228T and C250T have been observed in 6.5% of meningiomas at diagnosis and 28% of those undergoing malignant histological progression [39]. Goutagny et al. described six meningiomas with *TERT* mutations, five of which were associated with malignant histological progression [40]. In this subset of patients, they found that the *TERT* promoter mutation was found in both the lower and higher-grade tumour, and in both *NF2*-mutated and *NF2*-wildtype tumours. We identified two patients with meningiomas with *TERT* promoter mutations. One patient had tumours that were *NF2*-mutated and one had tumours in which no *NF2* mutation was detected; both were convexity meningiomas and both progressed from Grade 2 to 3. Interestingly in patient A, we were unable to detect the *TERT* promoter mutation in the progressed tumour, and of note this was resected after radiotherapy. Patient B (*NF2*-mutated tumours) on the other hand, had *TERT* promoter and *NF1* mutations in all tumour grades, suggesting that a *TERT* promoter mutation is not an oncogenic driver, from Grade 2 to 3, as it was also present in the Grade 2 tumour. However, *TERT* promoter mutations were not detected in any Grade 1 tumours that progressed in grade, thus *TERT* promoter mutations may play a role in progression beyond Grade 1.

We identified a *PTPN11* variant in all tumours in one patient. Recurrent germline *PTPN11* mutations in exons 3 and 13 are associated with Noonan syndrome (NS) characterised by multiple congenital anomalies. Individuals with NS have an increased risk of cancer. On review of the literature, we identified 27 patients with NS and *PTPN11* mutations diagnosed with brain tumours; dysembryoplastic neuroepithelial tumour (n = 11), oligodendroglioma (n = 2), medulloblastoma (n = 1), low grade glioma (n = 9) and high-grade glioma (n = 4). There are no reports of a patient with NS with a *PTPN11* mutation and a Grade 2 meningioma. Similarly, we identified a *TSC*2 variant in all tumours from a patient in whom no *NF2* mutations were found and without known tuberous sclerosis (TS). Cortical tubers, subependymal nodules and subependymal giant cell astrocytoma are characteristic intracranial lesions of TS. There has been one case of a meningioma reported in a patient with confirmed TS and one in a patient with suspected TS and a family history of neurofibromatosis type 2 [41, 42].

Four patients in our cohort had tumours with *NF2* mutations, none of our patients were known to have *NF2* germline mutations. The *NF2* mutations were present in the pre- and post-progression meningioma from each patient, of note, patient G had three different *NF2* mutations in four tumours suggesting separate mutational evolution. Our study confirmed that *NF2* mutations define a distinct subclass of non-skull base meningiomas that have a predilection to progress in grade. A 2020 study, suggested that high grade/progressive meningiomas be divided into three sub-groups; *NF2*-associated canonical group, *NF2*-agnostic group and *NF2*-exclusive group [43]. The most common was the *NF2*-associated canonical group, which as confirmed in our study, also showed loss of chromosome 1p. Patients in our cohort with tumours with *NF2* mutations showed frequent CNAs with similar patterns across patients, in contrast, tumours with no *NF2* mutation detected showed less frequent CNAs. In a study on 383 Chinese patients, that included 331 Grade 1, 46 Grade 2 and 6 Grade 3 tumours, it is was shown that more CNAs were found in higher grade tumours, recurrent lesions, tumour diameter over 4.3 cm and samples from male patients [44]. In particular, CNLs of 1p31.3 and 1p34.3, were commonly seen in high-grade and recurrent meningiomas. Cytogenetic analysis with fluorescence in-situ hybridisation (FISH) has shown deletions in 22, 1p and 14q in a subset of eleven meningiomas with grade progression [45]. Our study shows that this loss of 1p is present in the pre-progressed and post-progressed tumour and is uniformly associated with *NF*2 mutated tumours. We also confirmed loss of 14q across all grades in two patients whose tumours were *NF2* mutant. In addition, in our cohort, tumours with *NF2* mutations were strongly associated with losses on chromosome 10 and CNAs on 2p, 3p and 4p. Of note, CNAs on 22q were present in tumours from six patients including two patients who had tumours with no *NF2* mutations detected, including a large copy number gain in region 22q containing *NF2* in patient F.

Tumours from patients F and H (tumours in which no *NF2* mutation was detected) shared similar patterns of CNAs, including an interesting combination of loss and high gain (x4) on chromosome 17q. This pattern of localised copy gain is suggestive of amplification of extra-chromosomal DNA (ecDNA). It has been shown that ecDNA is associated with oncogene amplification and poor outcome, and has been confirmed to be especially prevalent in glioblastoma [46]. A previous study examining the 17q chromosomal region using FISH in seven patients with recurrent meningiomas confirmed a deletion in the 17q chromosomal region in 90.1% of tumours [47].

Eight patients in our cohort received radiation in the study collection period, with seven of these patients having subsequent tumour resections with included sequenced tissue. There is a lack of data in the literature specific to meningiomas investigating mutational profiles and CNAs pre- and post-radiation. In one study, meningiomas treated with adjuvant radiation exhibited a significantly higher burden of CNAs than radiation-induced or non-irradiated meningiomas [48]. Tumours from patient C exhibited a distinct increased burden of CNAs on progression to Grade 3. However, this increased burden of CNAs also coincided with radiation treatment, making it difficult to interpret the temporal relationship. In contrast, for patient G, the increased frequency of CNAs showed no correlation with radiation timing, as was the case for the other patients with less frequent CNAs. It is therefore possible that the increased frequency in CNAs seen in patient C is related to grade progression rather than radiation therapy.

## Limitations

This study has taken advantage of a unique cohort of ten patients with long term follow up and banked tumour tissue, but sequencing was performed on FFPE tissue, which limited our ability to perform concurrent whole exome sequencing. Matched germline DNA was also not available for sequencing, inhibiting our capacity to unequivocally claim that all detected mutations are somatic.

Because of the resolution of the TruSight Oncology 500 (TSO500) panel we have focussed on large-scale changes in our reported results, rather than prioritising small-scale copy number alterations. Tumours were classified according to the 2016 WHO criteria, which has since been updated in 2021. The 2021 WHO classification endorses molecular biomarkers to support classification and grading of meningiomas, however, of note, meningioma subtypes and CNS WHO grades remain primarily based on histologic criteria. Our findings suggest that spatial sampling to analyse spatial genetic heterogeneity will be useful for future work. However, of note, to assess the robustness of our methods four tumours were sampled and analysed twice. Analysis of these replicates suggests that they were sampling the same population of tumour cells.

Additionally, we acknowledge that our sample size is small, but it is the largest dataset of its kind due to time taken to collect tumour tissue over multiple years with consistent follow up. Therefore, considering the lack of similar publicly available datasets, including matched samples, this study provides a valuable resource for the scientific community. We foresee future prospective cohorts requiring international collaborations of datasets to combine data to perform larger analysis, with the ultimate aim to identify an aggressive meningioma at first resection and therefore guide subsequent treatment and surveillance. Future directions would also include sequencing a cohort of matched patients with meningiomas that recurred but did not progress in grade from matched anatomical locations for direct comparison. In addition, it would also be useful to include RNA sequencing to identify fusions and look for differential gene expression.

Banked tissue included in this study has been collected at multiple time points from the same patient spanning over a decade. Timing to progression and recurrence and patient age varies in each case, as does the degree of resectability, this therefore results in minor inconsistency in the treatment paradigm across patients. However, the universal principle of repeat resection at progression on surveillance imaging and adjunct radiation for all WHO 3 tumours was adhered to. Nonetheless, we were unable to perform a uniform analysis of patients with absolute consistent treatment regimens.

## Conclusion

Our study confirms that meningiomas that progress in WHO grade, in general, have a mutational profile that is already detectable in the pre-progressed tumour. This is consistent with the limited data available in the literature, including Viaene et al. that showed that the RNA profiles of four progressive grade 1 tumours clustered closely to their respective secondary grade 2 tumours, suggesting that progressive tumours mostly resemble their primary. Therefore, detection of these grade progression susceptible profiles may be useful to predict grade progression and modify treatment and follow-up accordingly, including timing of adjuvant therapy and frequency of surveillance.

Mutations in *SETD2*, *TP53*, *TERT* promoter and *NF2* were not consistently present across recurrent tumours. Although Mellai et al. reported the association of the TERT promoter mutations to histologic grade [49]. TERT promoter mutations have not been found to be significantly associated with the recently described molecular subtypes [15, 50]. Overall, the identification of genetic heterogeneity suggests that other mechanisms (such as epigenetic or structural variants) may also influence tumour grade progression. The amplification of various genes across tumours suggests that somatic CNAs may precede driver mutations resulting in heterogeneity in mutational profiles.

## Electronic supplementary material

Below is the link to the electronic supplementary material.

Supplementary Material 1 Mutations identified utilising the Illumina TSO500 NGS panel across all tumours sequenced. Patients labelled A-J. Mutation identified (Y). Mutation not identified (N).

Supplementary Material 2 Illumina TSO500 NGS Gene List

Supplementary Material 3 Estimated effect and significance for the association analysis between genomic aberrations and the genomic-wide load of gene copy number alterations.

## Data Availability

The datasets generated and/or analysed during the current study are available in The European Genome-phenome Archive (EGA): https://ega-archive.org/studies/EGAS00001006585. Accession number EGAS00001006585.

## List of abbreviations

WHO: World Health Organisation
HREC: Human Research Ethics Committee
CNA: Copy Number Alterations
CNS: Central Nervous System
ACCORD: Australian Comprehensive Cancer Outcomes and Research Database
ECOG: Eastern Cooperative Oncology Group
STR: Subtotal Resection
GTR: Gross Total Resection
FFPE: Formalin fixed paraffin embedded
HE: Hematoxylin and Eosin
UMI: Unique Molecular Identifier
SNVs: Single Nucleotide Variants
SV: Structural Variants
VAF: Variant Allele Fraction
BAF: b-allele-frequency
SNPs: Single Nucleotide Polymorphisms
LFC: Log-fold-Change
HET: Heterozygous
HOM: Homozygous
LOH: Loss of Heterozygosity
GMM: Gaussian mixture model
BIC: Bayesian information criteria
NGS: Next-generation sequencing
TS: Tuberous Sclerosis
FISH: Fluorescence in-situ hybridisation
ecDNA: extra-chromosomal DNA
TSO500: TruSight Oncology 500
RTx: Radiotherapy
