# Supplementary material for: Somatic mutation landscape in a cohort of meningiomas that have undergone grade progression
Source: BMC Cancer. 2023 Mar 7;23:216. doi: 10.1186/s12885-023-10624-9 (PMC9990218; doi:10.1186/s12885-023-10624-9)
Supplement: Supplementary file 1 — Supplementary Material 1 Mutations identified utilising the Illumina TSO500 NGS panel across all tumours sequenced. Patients labelled A-J. Mutation identified (Y). Mutation not identified (N). [file 12885_2023_10624_MOESM1_ESM.docx]

**Supplementary Data 1**

|  | **Tumour One** | **Tumour Two** | **Tumour Three** | **Tumour Four** | **Tumour Five** | **Tumour Six** | **Tumour Seven** | **Tumour Eight** | **Tumour Nine** | **Tumour Ten** |
| --- | --- | --- | --- | --- | --- | --- | --- | --- | --- | --- |
| Patient A | A1 | A3 | A2 |  |  |  |  |  |  |  |
| WHO Grade | 2 | 2 | 3 |  |  |  |  |  |  |  |
| Location | Convexity | Convexity | Convexity |  |  |  |  |  |  |  |
| Operation Date | May 2005 | September 2006 | March 2011 |  |  |  |  |  |  |  |
| Radiation |  | November 2006 |  |  |  |  |  |  |  |  |
| *TERT* promoter | Y | Y | N |  |  |  |  |  |  |  |
| *ERCC1* | Y | Y | Y |  |  |  |  |  |  |  |
| Patient B | B1 | B2 | B3 |  |  |  |  |  |  |  |
| WHO Grade | 2 | 2 | 3 |  |  |  |  |  |  |  |
| Location | Convexity | Convexity | Convexity |  |  |  |  |  |  |  |
| Operation Date | April 2000 | January 2002 | January 2003 |  |  |  |  |  |  |  |
| Radiation |  | April 2002 |  |  |  |  |  |  |  |  |
| *NF2:p.Phe94Ter* | Y | Y | Y |  |  |  |  |  |  |  |
| *TERT promoter* | Y | Y | Y |  |  |  |  |  |  |  |
| *SPTA1* | Y | Y | Y |  |  |  |  |  |  |  |
| *NF1* | Y | Y | Y |  |  |  |  |  |  |  |
| Patient C | C3/4 | C1 | C2 | C5 | C6 | C7 |  |  |  |  |
| WHO Grade | 1 | 2 | 3 | 3 | 3 | 3 |  |  |  |  |
| Location | Parasagittal | Parasagittal | Parasagittal | Parasagittal | Parasagittal | Parasagittal |  |  |  |  |
| Operation Date | April 1997 | February 2000 | September 2006 | April 2007 | September 2007 | December 2007 |  |  |  |  |
| Radiation |  | March 2000 |  |  |  |  |  |  |  |  |
| *NF2:p.Arg57Ter* | Y | Y | Y | Y | Y | Y |  |  |  |  |
| *POLE* | Y | N | N | N | N | N |  |  |  |  |
| Patient D | D1 | D4 | D2 | D5 | D6 | D3/D7 | D9 |  |  |  |
| WHO Grade | 1 | 1 | 3 | 3 | 3 | 3 | 3 |  |  |  |
| Location | Sphenoid Wing | Sphenoid Wing | Sphenoid Wing | Sphenoid Wing | Sphenoid Wing | Sphenoid Wing | Sphenoid Wing |  |  |  |
| Operation Date | December 2000 | November 2002 | March 2003 | October 20008 | March 2010 | November 2010 | November 2011 |  |  |  |
| Radiation |  |  | March 2003 |  |  |  |  |  |  |  |
| *SETD2* | Y | N | N | Y | Y | Y | Y |  |  |  |
| *NAB2-STAT6 Inversion* | Y | Y | Y | Y | Y | Y | Y |  |  |  |
| *PIK3C2B* | Y | Y | Y | Y | Y | Y | Y |  |  |  |
| *DNMT3B* | Y | Y | Y | Y | Y | Y | Y |  |  |  |
| *ABRAXAS1* | Y | Y | Y | Y | Y | Y | Y |  |  |  |
| *IRF4* | Y | Y | Y | Y | Y | Y | Y |  |  |  |
| Patient E | E1 | E2 | E3 |  |  |  |  |  |  |  |
| WHO Grade | 1 | 1 | 2 |  |  |  |  |  |  |  |
| Location | Convexity | Convexity | Convexity |  |  |  |  |  |  |  |
| Operation Date | August 2003 | August2007 | March 2011 |  |  |  |  |  |  |  |
| Radiation |  |  |  |  |  |  |  |  |  |  |
| *NF2:p.Lys332SerfsTer14* | Y | Y | Y |  |  |  |  |  |  |  |
| *CREBBP* | Y | Y | Y |  |  |  |  |  |  |  |
| *MST1* | Y | Y | Y |  |  |  |  |  |  |  |
| *PNRC1* | Y | Y | Y |  |  |  |  |  |  |  |
| Patient F | F1 | F3 | F2/4 |  |  |  |  |  |  |  |
| WHO Grade | 1 | 1 | 2 |  |  |  |  |  |  |  |
| Location | Sphenoid Wing | Sphenoid Wing | Sphenoid Wing |  |  |  |  |  |  |  |
| Operation Date | May 1997 | 1998 | January 2006 |  |  |  |  |  |  |  |
| Radiation | October 1997 |  |  |  |  |  |  |  |  |  |
| *TSC2* | Y | Y | Y |  |  |  |  |  |  |  |
| Patient G | G1 | G4 | G5 | G6 | G7 | G2 | G3 |  |  |  |
| WHO Grade | 1 | 1 | 1 | 1 | 1 | 1 | 2 |  |  |  |
| Location | Falcine | Falcine | CP Angle | Falcine | Falcine | Falcine | Falcine |  |  |  |
| Operation Date | July 1989 | September 1994 | October 1994 | October 1995 | October 1996 | June 2000 | June 2003 |  |  |  |
| Radiation |  |  |  |  | 1999 |  |  |  |  |  |
| *NF2:p.Ala323ProfsTer23* | N | N | N | N | N | Y | N |  |  |  |
| *NF2:p.Glu342Ter* | N | Y | N | N | Y | N | N |  |  |  |
| *NF2:p.Glu362ArgfsTer13* | N | N | Y | N | N | N | N |  |  |  |
| *NF2:p.Ala367Gly* | N | N | Y | N | N | N | N |  |  |  |
| *SF3B1* | Y | Y | Y | Y | Y | Y | Y |  |  |  |
| Patient H | H1 | H2 |  |  |  |  |  |  |  |  |
| WHO Grade | 1 | 2 |  |  |  |  |  |  |  |  |
| Location | Convexity | Convexity |  |  |  |  |  |  |  |  |
| Operation Date | June 2000 | March 2006 |  |  |  |  |  |  |  |  |
| Radiation |  |  |  |  |  |  |  |  |  |  |
| *IFNGR1* | Y | Y |  |  |  |  |  |  |  |  |
| Patient I | I1/I3 | I4 | I2/I5 |  |  |  |  |  |  |  |
| WHO Grade | 1 | 1 | 2 |  |  |  |  |  |  |  |
| Location | Sphenoid Wing | Sphenoid Wing | Sphenoid Wing |  |  |  |  |  |  |  |
| Operation Date | December 2006 | August 2008 | March 2012 |  |  |  |  |  |  |  |
| Radiation |  |  | March 2012 |  |  |  |  |  |  |  |
| *PTPN11* |  | Y | Y |  |  |  |  |  |  |  |
| *PIK3CA* |  | Y | Y |  |  |  |  |  |  |  |
| *TRAF7* |  | Y | Y |  |  |  |  |  |  |  |
| *LATS2* |  | Y | Y |  |  |  |  |  |  |  |
| *GEN1* |  | Y | Y |  |  |  |  |  |  |  |
| *LRP1B* |  | Y | Y |  |  |  |  |  |  |  |
| Patient J | J1 | J2/J5 | J3 | J6 | J7 | J8 | J4 | J9 | J10 | J11 |
| WHO Grade | 2 | 2 | 3 | 3 | 3 | 3 | 3 | 3 | 3 | 3 |
| Location | Sphenoid Wing | Sphenoid Wing | Sphenoid Wing | Sphenoid Wing | Sphenoid Wing | Spinal Cord | Sphenoid Wing | CP Angle | CP angle | Spinal Cord |
| Operation Date | July 1997 | July 1998 | June 2004 | August 2006 | March 2007 | June 2007 | January 2008 | April 2008 | June 2008 | June 2009 |
| Radiation |  | September 2002 |  |  |  |  |  |  |  |  |
| *TP53* | N | N | N | N | N | Y | Y | Y | Y | Y |
| *NAB2-STAT6 Inversion* | Y | Y | Y | Y | Y | Y | Y | Y | Y | Y |
| *SPTA1* | Y | Y | Y | Y | Y | Y | Y | Y | Y | Y |
| *SLX4* | Y | Y | Y | Y | Y | Y | Y | Y | Y | Y |
| *CDK12* | Y | Y | Y | Y | Y | Y | Y | Y | Y | Y |
| *NKX3-1* | Y | Y | Y | Y | Y | Y | Y | Y | Y | Y |
