# Supplementary figures and images for: Somatic mutation landscape in a cohort of meningiomas that have undergone grade progression
Source: BMC Cancer. 2023 Mar 7;23:216. doi: 10.1186/s12885-023-10624-9 (PMC9990218; doi:10.1186/s12885-023-10624-9)

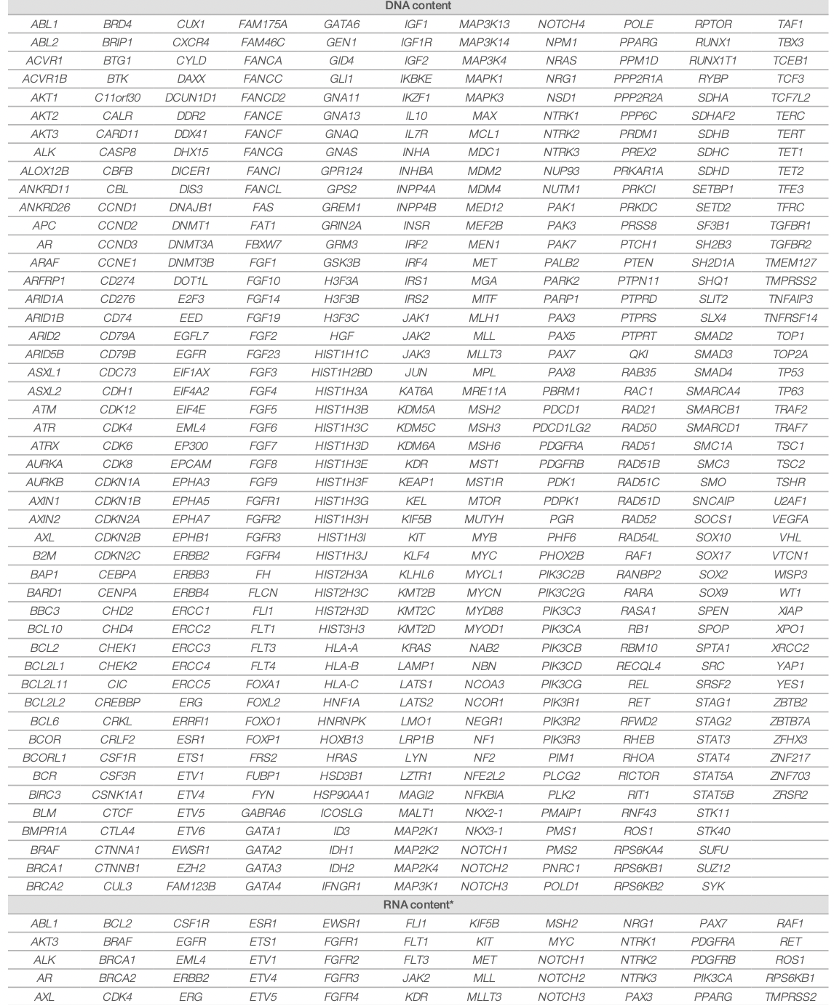

Supplement: Supplementary file 2 — Supplementary Material 2 Illumina TSO500 NGS Gene List [file 12885_2023_10624_MOESM2_ESM.png]
