# Supplementary material for: Somatic mutation landscape in a cohort of meningiomas that have undergone grade progression
Source: BMC Cancer. 2023 Mar 7;23:216. doi: 10.1186/s12885-023-10624-9 (PMC9990218; doi:10.1186/s12885-023-10624-9)
Supplement: Supplementary file 3 — Supplementary Material 3 Estimated effect and significance for the association analysis between genomic aberrations and the genomic-wide load of gene copy number alterations. [file 12885_2023_10624_MOESM3_ESM.docx]

Supplementary Data 3

| Genomic Feature | Effect genomic feature present TRUE | P value (adjusted BH) |
| --- | --- | --- |
| NF2 | 1.546414927 | 1.80E-14 |
| NAB2-STAT6 | -3.007561872 | 1.97E-08 |
| SETD2 | -2.543634662 | 0.05640922894 |
| TSC2 | -1.82628575 | 0.06635826547 |
| PTPN11 | 0.3326760173 | 1 |
| TERTp | -0.4326675884 | 1 |
| TP53 | -15.8656588 | 1 |

Estimated effect and significance for the association analysis between genomic aberrations and the genomic-wide load of gene copy number alterations.
